# Learning and switching between stimulus-saccade associations in Parkinson’s disease^☆^

**DOI:** 10.1016/j.neuropsychologia.2013.03.026

**Published:** 2013-06

**Authors:** Timothy L Hodgson, Petroc Sumner, Dimitra Molyva, Ray Sheridan, Christopher Kennard

**Affiliations:** a School of Psychology University of Lincoln, Brayford Pool, Lincoln LN6 7TS, United Kingdom; b School of Psychology, University of Cardiff, Tower Building, Park Place, Cardiff CF10 3AT, United Kingdom; c Department of Pharmacology, School of Medicine, Aristotle University of Thessaloniki, Thessaloniki, Greece; d Department of Geriatric Medicine, Royal Devon and Exeter Hospital, Barrack Road, Exeter, Devon EX2 5DW, United Kingdom; e Nuffield Department of Clinical Neurosciences, Level 6, West Wing, John Radcliffe Hospital, Oxford OX3 9DU, United Kingdom

**Keywords:** Eye movements, Basal ganglia, Task switching, Oculomotor, Cognitive

## Abstract

Making flexible associations between what we see and what we do is important for many everyday tasks. Previous work in patients with focal lesions has shown that the control of saccadic eye movements in such contexts relies on a network of areas in the frontal cerebral cortex. These regions are reciprocally connected with structures in the basal ganglia although the contribution of these sub-cortical structures to oculomotor control in complex tasks is not well understood. We report the performance of patients with idiopathic Parkinsons disease (PDs) in a test which required learning and switching between arbitrary cue-saccade rules. In Experiment 1 feedback was given following each response which reliably indicated which of the two possible rules was correct. PDs were slower to learn the first cue-saccade association presented, but did not show increased error or reaction time switch costs when switching between two rules within blocks. In a follow up experiment the feedback given by the computer was adjusted to be probabilistic such that executing a response based upon the “correct” rule only resulted in positive feedback on 80% of trials. Under these conditions patients were impaired in terms of response latencies and number of errors. In all conditions PDs showed multi-stepping/hypometria of saccades consistent with a motoric deficit in executing actions based on cognitive cues. The findings are consistent with a role for the nigrostriatal dopamine system in the reinforcement of saccade-response-outcome associations. Intact performance of PDs when associations are not stochastically reinforced suggests that striatal learning systems are complemented by cognitive representations of task rules which are unaffected in the early stages of PD.

## Highlights

• We examine how People with Parkinsons learn rules linking visual cues and saccades.
• Patients were slower to learn single colour-saccade direction rule mappings.
• Patients made increased errors but no increase in rule “switch costs”.
• The deficits related to dopamine and cortico-striatal circuitry.
• The findings have relevance for understanding deficits in everyday tasks.

## 1 Introduction

In order to perform everyday tasks such as cooking a meal, changing a light bulb or driving a car appropriate task rules need to be learned and maintained in working memory and then transformed into appropriate motor (including oculomotor) output to achieve task goals (Hodgson & Golding, 2003; Land & Furneax, 1997). One potential neural substrate of this ability are so-called cortico-striatal loops linking structures in the prefrontal cortex with the basal ganglia. In this paper we describe the performance of mild to moderately affected people with Parkinsons disease (PDs) in a series of tasks involving learning and switching between stimulus-saccade rules. Deficits might be predicted in the task in PDs on at least two counts. Firstly, dopamine is known to play an important modulatory role in cortico-striatal circuitry (Chakravarthy, Joseph, & Bapi, 2010; Frank, 2005; Graybiel, 1997; Schultz, Dayan, & Montague, 1997). Secondly, patients with focal frontal cortex damage, schizophrenia and other types of striatal pathology have already been shown to have performance deficits in the same or similar saccadic tasks (Golding, Danchaivijitr, Hodgson, Tabrizi, & Kennard, 2006; Hodgson et al., 2007; Huddy, Hodgson, Ron, Barnes, & Joyce, 2011; Husain, Parton, Hodgson, Mort, & Rees, 2003; Parton et al., 2007; Robert et al., 2009).

Post-mortum studies have confirmed that dopaminergic cell death in the substantia nigra pars compacta is consistently observed across patients who have died with idiopathic Parkinsons Disease (PD). Whilst adjacent regions of the substantia nigra (e.g. pars reticulata) can also be affected, cell death in these areas occurs to a more variable extent across individuals (Damier, Hirsch, Agid, & Graybiel, 1999). It is the reduction in the dopaminergic input from the substantia nigra pars compacta into the striatum that initially gives rise to the motoric symptoms of the disease. Post-mortum studies have shown that dopamine loss is maximal within the putamen portion of the striatum during early stages (Kish, Shannak, & Hornykiewicz, 1988), whilst the caudate nucleus is relatively unaffected, although significant depletion is found even in early stage patients within the head of the caudate. Most recently, in vivo magnetic resonance diffusion tensor imaging (DTI) studies have confirmed that the head of the caudate is connected to the medial, ventral and dorso-lateral prefrontal cortex as well as the frontal pole and pre-supplementary motor area (Lehericy et al., 2004). All of these cortical regions have been strongly implicated in cognitive or cognitive-motor functions. One commonly held interpretation of this neuro-anatomical organization is that segregated cortico-striatal “loops” maintain the principles of functional specialization of the overlying cortex such that different loops subserve specialist motor, oculomotor and cognitive functions (Alexander & Crutcher, 1990).

Although there is now overwhelming evidence for the existence of mild cognitive deficits in the early stage of PD the exact nature of these impairments and how they relate to the normal functioning of basal ganglia circuits is still debated (Grahn, Parkinson, & Owen, 2008; Middleton & Strick, 2000). One aspect of the Parkinsonian cognitive syndrome is assumed to be a lack of cognitive flexibility as indexed by tests of attentional set-shifting and task switching (Bowen, Kameinny, Burns, & Yahr, 1975; Cools, Van den Bercken, Horstink, van Spaendonck, & Berger, 1984; Downes et al., 1989; Flowers, & Robertson, 1985; Owen et al., 1992, 1993). Many tasks of this type require several component cognitive processes including working memory, shifting attention between stimulus categories and error monitoring as well as switching between stimulus-response mappings (the classic example being the Wisconson Card sorting test, Grant & Berg, 1948). Cools, Barker, Sahakian, and Robbins (2001a) attempted to disentangle attentional set from other task demands and report increased task switch costs in PDs even when rules were explicitly cued and patients did not have to learn rules by trial and error (i.e. no working memory or error monitoring demands). The deficit was particularly marked under conditions for which each response contingent stimulus was associated with two possible responses depending upon the current task rule mapping (so called “bi-valent” stimuli). However, an earlier study using less severely affected PD patients and a near identical procedure did not report a significant impairment in task switching (Rogers et al., 1998).

The present study examines learning and switching between stimulus-saccade mappings in PD using a rule switching task that has been studied extensively in other patient groups. Although no previous studies have examined PDs performance in this task, their eye movements during more simple tests have been extensively studied and a number of consistent findings are apparent. For simple stimulus driven movements (so-called reflexive or “pro” saccades) normal amplitude and response latency are typically found in PD (e.g. Briand, Strallow, Hening, Poizner, & Sereno, 1999; Fukushima, Fukushima, Miyasaka, & Yamashita, 1994; Hodgson, Dittrich, Henderson, & Kennard, 1999; Lueck, Tanyeri, Crawford, Henderson, & Kennard, 1990; Mosimann et al., 2005). However, when movements are executed under memory-guided conditions saccades become markedly hypometric, possibly due to disruption of a functionally specific oculomotor cortico-striatal loop via the caudate (Briand et al., 1999; Crawford, Henderson, & Kennard, 1989; Hodgson et al., 1999; Middleton & Strick, 2000). Similar abnormalities in movement kinematics have been noted for skeletomotor movements when responding under open loop conditions (Alberts, Saling, Adler, & Stelmach, 2000; Jackson, Jackson, Harrison, Henderson, & Kennard, 1995; Ketcham, Hodgson, Kennard, & Stelmach, 2003; Rand & Stelmach, 2000; Swinnen, Steyvers, VanDenBergh, & Stelmach, 2000). A general theory of striatal function which is consistent with these findings is that the caudate nucleus mediates “cognitive-motor” as opposed to sensori-motor transformations when actions have to be generated on the basis of an internally represented goal (Ketcham, Hodgson, Kennard, & Stelmach, 2003; Levy, Friedman, Davachi, & Goldman-Rakic, 1997; Postle, & D'Esposito, 1999). Saccadic hypometria under memory-guided conditions may therefore reflect a problem in executing actions based upon cognitive representations, rather than an impairment in spatial working memory per se.

In the rule switching task participants learn a rule linking a central cue with a saccade to either the left or the right (Hodgson, Golding, Molyva, Rosenthal, & Kennard, 2004). The rule can reverse at different points in the task as indicated by the positive/negative feedbacks presented following each response. Interestingly, healthy participants often make saccade errors following a rule change, although these are usually followed by rapid corrective movements (the computer only presents feedback when a fixation longer than 800 ms has been made at one of the response locations, thus allowing corrective movements to be made). The occurrence of such corrected errors in the task is found to be greatly increased following supplementary eye field damage (Husain et al., 2003). In contrast patients with inferior frontal cortex damage make significantly increased actual (uncorrected) errors when switching between rules (Hodgson et al., 2007). Saccadic latencies are also found to be much slower to the location at which a negative feedback has been presented on a preceding trial and this negative priming like effect (Tipper, 1985) is found to be reduced following orbitofrontal cortex damage (Hodgson, Mort, Chamberlain, O'Neill, & Kennard, 2002; Hodgson et al., 2004).

Given the previous findings in frontal patients and the known pathology of cortico-striatal loops in PD a number of performance deficits in the rule switching task might be predicted even in mild to moderately affected patients, with the exact nature of the expected deficits depending upon how one views the functional organization of cortico-striatal circuits. For example, if the anterior striatum forms a key functional component of the network via which rules are monitored and maintained within working memory along with structures in the frontal cortex then PDs should show very similar deficits to lateral prefrontal lesion patients, making increased errors in the task due to a failure to maintain in mind the current rule. Consistent with this idea, a recent fMRI study has suggested that preparatory set activity in frontal cortex is disrupted during oculomotor task performance in PD (Cameron et al., 2012). Alternatively, if the caudate nucleus simply forms a conduit via which goal information is transformed from working memory into action then patients should only show impairments in more motoric measures (e.g. hypometria or saccadic corrections). Finally, if PDs have a specific deficit under conditions for which two competing stimulus-response associations have to be selected between then they should show impairments specific to trials following a change in rule mappings, where such response/rule conflict should be maximal.

## 2 Experiment 1

### 2.1 Methods

#### 2.1.1 Subjects/patients

##### 2.1.1.1 Control group

The control group comprised 15 participants selected to match as closely as possible the age and gender of the patient group. Eight were male and 7 were female. Ages ranged from 50 to 70 years with a mean age of 63 years.

##### 2.1.1.2 Patient group

Nineteen patients with mild to moderate idiopathic PD participated in Experiment 1. The mean age of the patient group was 63 years ranging from 48 to 74 years. Patients were recruited from a volunteer database at Charing Cross Hospital, London (13 patients) as well as via a movement disorders clinic at the Royal Devon and Exeter Hospital (6 patients). Patient details are given in Table 1 including current medication, Hoen and Yahr (Hoehn & Yahr, 1967) and Webster score assessments (Webster, 1968). The Webster assessment is almost identical to the motoric sub-test of the unified parkinsons disease rating scale (Fahn & Elton, 1987) and approximate equivalent UPDRS III scores (based upon a linear rescaling factor of 1.43) are given in the table. All patients were taking dopaminergic medication at the time of testing. Research was approved initially by the Riverside mental health ethics committee (Charing Cross Hospital patients) and then the South West regional committee of the National NHS research ethics system (Exeter patients) as well as via the School of Psychology, University of Exeter research ethics committee.

Both patient and control groups completed the National Adult Reading Test (NART), mini-mental and digit span test prior to oculomotor testing. Statistical comparison of age and performance measures between groups via independent two sample *t*-tests showed no statistically significant differences in any factor.

#### 2.1.2 Eye movement recording

Eye movements were recorded using an Eyelink Eye Tracker (SR Research Ltd.), a video based pupil tracker, with head movement compensation system sampling at 250 Hz. Subjects were seated at a comfortable viewing distance in front of the display monitor approximately 60 cm from the computer screen. Pupil position was monitored via two miniature infrared CCD video cameras mounted on an adjustable headband. Subjects were instructed to keep head movements to a minimum and no active restraint of head movements was required to obtain sufficiently accurate gaze position recordings. Eye movements were visualized off-line, saccades were identified and artifacts removed using custom software programs developed within the LabVIEW visual programming environment using a saccade detection criterion threshold of 30° per second for at least 3 consecutive samples. Approximately 4% of trials had to be excluded from analysis due to gross artefacts or signal loss arising from eye-lid interference or blinks.

#### 2.1.3 Procedure

##### 2.1.3.1 General

Experiment 1 comprised three blocks of trials, the first requiring learning of a single colour-saccade rule (*Simple Associative task*) the other two requiring switching between two conflicting rules (*Rule Switching task*). Following completion of consent forms, pencil and paper cognitive and disease assessments, eye tracker setup and calibration (see above), patients completed the eye tracking tasks. In addition to the rule switching tasks, patients and controls typically also completed a number of other oculomotor tests during the testing session, the results of which are not reported in the current paper. Rule learning and switching tasks were completed in a fixed order with the Simple Associative (single rule) blocks always presented prior to the switching blocks in Experiments 1 and 2. The 1st rule presented in the switching blocks was always the same as the rule the patients had learned in the preceding Simple Associative block. Three of the patients and 3 of the control group also completed Experiment 2 (see below) at a later testing session date.

##### 2.1.3.2 Simple associative task

This was identical to the rule reversal task described below except that the association between cue and correct response remained constant throughout a block of 60 trials. Participants were instructed that the colour of the cue indicated whether they should make a response to either the left or right response box and that they had to work out which colour corresponded to which response.

##### 2.1.3.3 Rule switching task

**Fig. 1:**
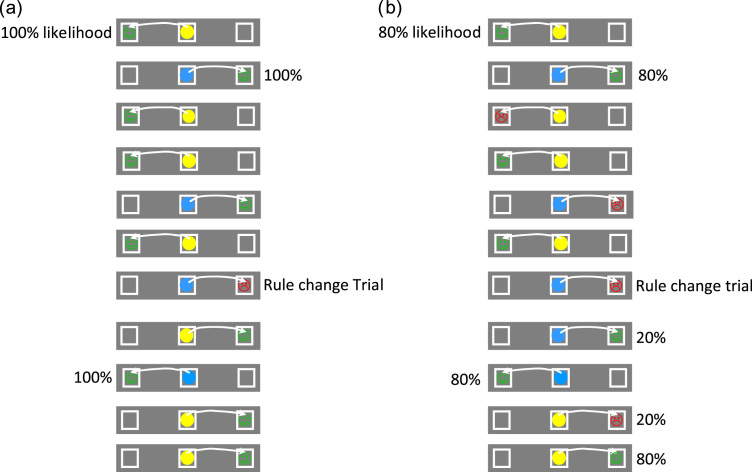
Schematic of typical trial sequence in the rule switching tasks. Arrows represent saccadic responses to left or right response box: (a) standard rule switching task (Experiment 1), in which positive/negative feedbacks reliably indicate the rule. (b) Probablistic rule switching task (Experiment 2) for which positive feedbacks occur with 80% likelihood when the optimal rule is used.

Three boxes, outlined in black on a grey coloured background, were presented in the centre and 9° to the left and right of the centre of a 22 in. colour monitor. Each box subtended 3° of visual angle. Every trial was triggered to start when the subject had been continuously fixating the central location for a period greater than 800 ms. Following this period, either a blue/red circle or a blue/yellow circle was displayed in the central box (the two colour sets being varied randomly across participants and each participant receiving only one colour set). The colour of the central cue instructed the subject whether to look left or right. The next fixation longer than 800 ms on either the left or the right box was taken as the subject's response on that trial, such that several eye fixations of shorter duration could be made before the subject made their final decision. Once the participant had selected one of the boxes in this manner, feedback was given to indicate if the choice was correct or incorrect in the form of a happy/sad face displayed within the selected box, accompanied by a high or low pitched tone (Fig. 1a). Subjects were made aware that the rule would reverse at several points during the test but that the first rule was always the same as that presented in the Simple Associative task block. Rule changes were indicated by unexpected errors. Each subject completed two blocks of 100 trials, comprising a maximum of 16 possible rule reversals. They were instructed to perform the task as quickly and as accurately as possible and to respond on the basis of the rule they knew to be correct at that time, without anticipating the occurrence of a rule change.

### 2.2 Results

#### 2.2.1 Simple Associative Task

**Fig. 2:**
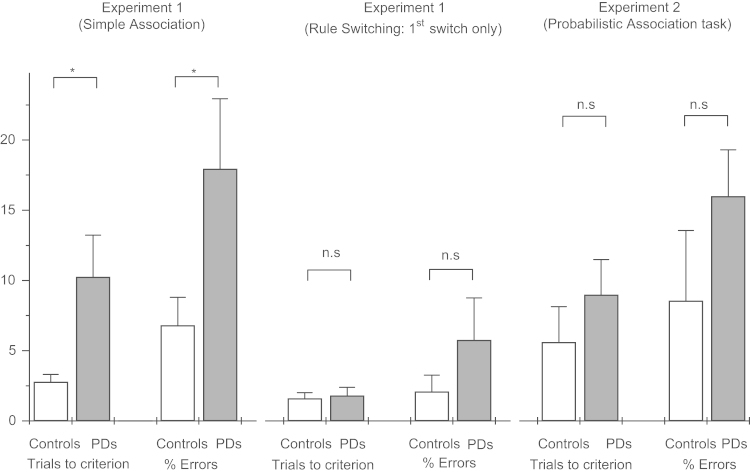
Errors and trials to learning criterion for non-switching blocks in Experiments 1 and 2 and the first rule change encountered in the rule switching condition of Experiment 1 (*Indicates significant difference between groups at *p*<0.05 or greater. n.s.=non-significant difference).

Independent samples 2-tailed *t*-tests were used to compare the main performance measures between PDs and healthy control participants. The point at which participants were deemed to have learned the rule was set as the start of the first run of 8 consecutive correct responses (chosen as this was the point at which the chances of a series of 8 random guesses conforming to the rule are less than 1/100). The number of trials taken to reach this criterion was found to be significantly increased for PDs relative to the control group (*t*=2.48, d.f.=32, *p<*0.025). There was also a significant difference in the number of response errors made by patients (*t*=2.18, d.f.=32, *p<*0.05) (Fig. 2). However, once criterion had been achieved there was found to be no significant difference in percentage errors between the two groups in the remainder of the block (*t*=0.99, d.f.=30). Patients also showed significantly increased mean saccade response latencies in the simple associative task (*t*=2.27, d.f.=32, *p<*0.05). Primary saccade amplitude on correct response trials (i.e. size of first saccade on each trial) was found to be significantly reduced for PDs relative to controls during the Simple Associative Task blocks (*t*=3.80, d.f=32, *p<*0.001) (mean gain of saccadic amplitude relative to target position PDs: 0.82+−0.03; Controls: 0.97+−0.024).

##### 2.2.2 Rule switching task

###### 2.2.2.1 Saccadic amplitude

Primary saccadic amplitude was compared across groups and relative to the trial position after rule switch using a 2-way ANOVA with Group (control/patient) and Trial after rule change (1–8) as factors. Results revealed a strongly significant effect of Group, with PDs showing significant hypometria relative to Control participants (*F*(1,30)=16.03, *p<*0.0001) (mean gain of saccadic amplitude relative to target position PDs: 0.83+−0.02; Controls: 0.96+−0.02), but there was no effect of Trial (*F*(7,210)=1.42, *p*=0.20) or interaction effect between Trial and Group (*F*(7,210)=1.33, *p*=0.35) on saccade amplitude.

###### 2.2.2.2 Saccadic latency

**Fig. 3:**
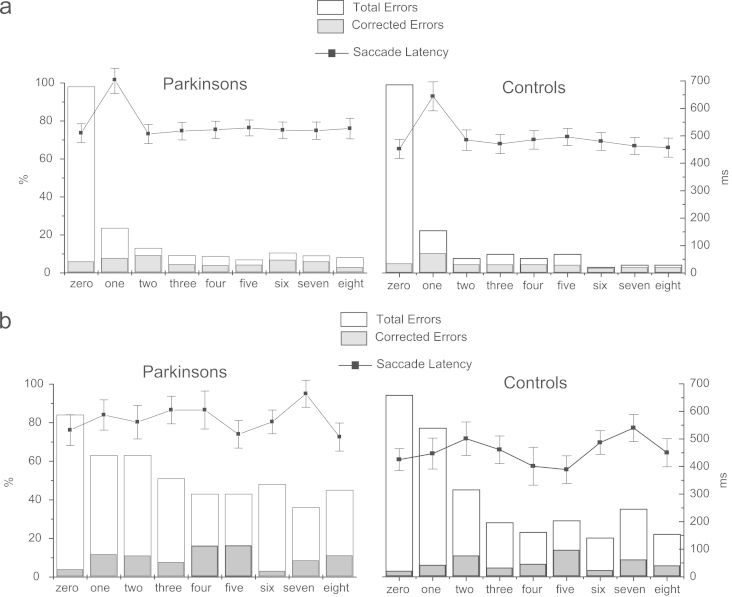
Errors and latencies in the two rule switching tasks plotted against trials after rule change for PDs and Controls. Trial “zero” represents the response on which the rule contingent post-response feedback was modified, such that “errors” represent responses correct according to the alternate rule: (a) Experiment 1 when feedback was 100% reliable. (b) Experiment 2 in which feedback only indicated the optimal rule with 80% validity.

The latency (i.e. reaction time) of the first saccade following cue onset was analysed using a 2-way Analysis of variance with Group (control/patient) and Trial after rule change as factors (trials 1–8). This revealed a significant main effect of Trial (*F*(7,210)=20.02, *p<*0.0001), but no main effect of Group (*F*(1,30)=1.52, *p*=0.23) or interaction between Group and Trial (*F*(7,210)=0.21, *p*=0.98). As expected based on previous studies (Hodgson et al., 2002b, 2004, 2007) latencies on the first trial following the rule change was greatly increased relative to all other trials (Fig. 3). However the size of the rule switch cost was not significantly greater in PDs compared to controls, neither was latency increased in patients overall.

Latency on the trial immediately preceding the feedback which signalled a rule change (labelled trial 0 in Fig. 3) was also analysed and showed no significant difference between groups (independent samples *t*-test: *t*=1.34, d.f.=30, *p*=0.19).

###### 2.2.2.3 Response errors

2-way Analyses of variance (ANOVAs) were carried out on error rates using Group (control/patient) and Trial after rule change (1–8) as factors. Errors were classified according to whether or not they were *corrected* with a secondary saccade prior to the feedback or *uncorrected* resulting in actual errors. The total number of errors (corrected plus uncorrected) was significantly increased on the first trial following a rule change (effect of Trial *F*(7,210)=8.92, *p<*0.0001), but there was no main effect of Group (*F*(1,30)=1.37, *p*=0.251) or interaction between Trial and Group (*F*(1,150)=0.88, *p*=0.52) (Fig. 3). Total errors trials on the trial for which participants received the feedback that indicated a rule change (i.e. trial 0), were also analysed and showed no significant difference between groups (independent samples *t*-test: *t*=0.646, d.f.=30, *p*=0.523).

For corrected errors a significant main effect of Trial was found (*F*(7,2100)=6.01, *p<*0.0001), but there was no main effect of Group (*F*(1,30)=0.43, *p*=0.52) or interaction between Group and Trial (*F*(7,210)=1.92, *p*=0.07). Corrected errors were also not significantly different between groups on rule change trials (independent samples *t*-test: *t*=0.43, d.f.=30, *p*=0.66).

For uncorrected errors (i.e. actual errors in which a negative feedback resulted) there was a significant effect of Trial (*F*(7,210)=7.26, *p<*0.001) and a main effect of Group (*F*(7,30)=5.87, *p*=0.022) but no significant interaction between Group and Trial (*F*(7,210)=0.83, *p*=0.56). The interaction between Trial and Group was also non-significant when the first trial following a rule change was compared to all subsequent trials (*F*(1,30)=0.51, *p*=0.48). Although there was a trend towards increased uncorrected errors later in a rule (see Fig. 3) direct means comparisons showed no evidence for a selective increase in error rates in the run up to a rule change (indicative of possible anticipatory rule changes), with no significant difference in error rates between groups for responses occurring more than 6 trials after a rule change (*t*=1.23, d.f.=30, *p*=0.225) and no significant difference in uncorrected errors between groups on trials immediately preceding a rule change (trial 0) (*t*=0.545, d.f.=30, *p*=0.59).

As the first rule change in the switching blocks always required participants to implement a reverse mapping to that which they had initially learned in the Simple Associative block, we also examined whether patients were impaired at acquiring this first example of the reversed rule relative to controls. This showed that PDs were not any worse than controls at acquiring the first instance of the reversed rule. PDs typically switched rule within 2 trials and did not make a significantly increased number of errors in this block relative to controls or other switches (Trials to Criterion PD: 1.82; Con: 1.60; *t*=1.01, d.f.=30, *p*=0.28; Errors PD: 6%; Con: 2%; *t*=0.30, d.f.=30, *p*=0.76) (Fig. 2).

###### 2.2.2.4 Negative priming effect

**Fig. 4:**
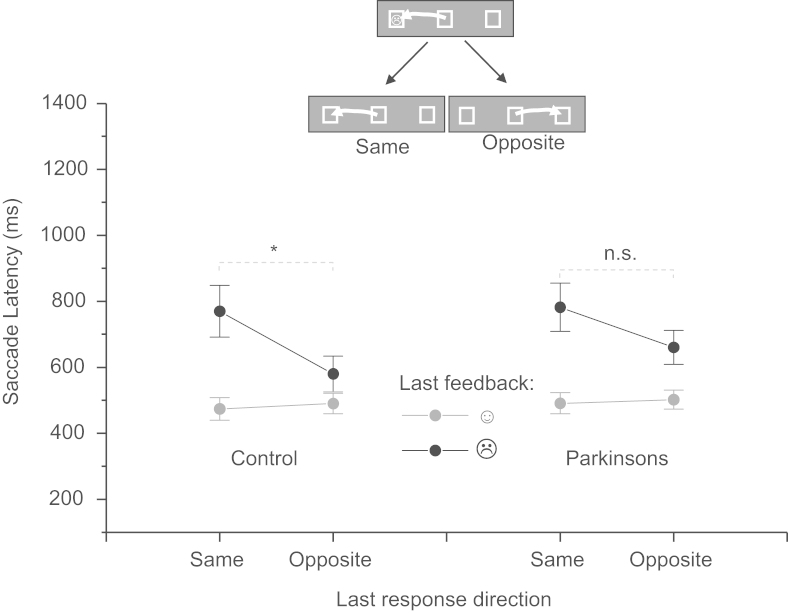
Reward related negative priming effect on saccade response latencies in patients and controls. Responses were significantly slowed when saccades are cued back to the same location at which a negative feedback had been presented on the previous trial (difference between same and different direction trials following a negative feedback *p<0.001; n.s. indicates non-significant).

Previous studies using the task have reported a marked slowing of response times for trials on which a participant is cued back to the location at which a negative feedback had been presented on the previous trial (Hodgson et al., 2002, 2004; Huddy et al., 2011). A 3-way ANOVA with Type of feedback on previous trial (positive/negative), Direction of response (same/different to last trial) and Group (control/patient) confirmed a significant main effect of Type (*F*(1,30)=44.56, *p<*0.0001), Direction (*F*(1,30)=11.56, *p<*0.005) and a 2-way interaction between Type and Direction on primary saccade latencies (*F*(1,30)=17.34, *p<*0.001). Latencies were significantly slower on trials following a negative feedback and particularly so for saccades directed back towards the same location at which the response had been directed on the preceding trial. Although the 3-way interaction with Group did not reach significance (*F*(1,30)=0.78, *p*=0.38) and an independent samples *t*-test directly comparing the size of the effect between groups showed no significant difference between PDs and Controls, examination of mean latencies revealed that the magnitude of the effect was reduced in PDs. Paired sample *t*-tests showed that for control subjects latencies were significantly elevated for different relative to same direction responses following a negative feedback (*t*=3.57, d.f.=14, *p<*0.001), whereas the difference was not significant for PDs (*t*=2.04, d.f.=16, *p*=0.06) (Fig. 4).

A bias to respond away from a negative feedback was also apparent in the direction of response errors. Errors were more likely to be made away from the location of a negative feedback but not a positive feedback (feedback Type by Direction interaction *F*(1,29)=17.16, *p<*0.0001). However, this interaction did not differ significantly in magnitude between the control and patient group.

#### 2.2.3 Correlations with disease severity

The goodness of fit of optimized linear and quadratic functions (see Cools, Barker, Sahakian, & Robbins, 2001b, 2001c; Rowe et al., 2008) were assessed for the relationship between Webster/UPDRS III scores and each of the following dependent measures: Total number of corrected and uncorrected errors in the task; Corrected and uncorrected error rates on the first versus second trial after a rule switch; Mean saccade latencies across all trials; Mean saccade latencies on the first versus second trial after rule change (i.e. latency switch cost); mean saccadic gain; The negative priming effect on saccade latency (defined as the latency difference between responses directed towards the same versus different response location to the last trial following a negative feedback).

Using the standard alpha value of 0.05 used in all the above analysis, the only performance measure which showed a relationship with disease severity was the magnitude of the negative priming effect. This showed a quadratic relationship with disease severity (*R*^2^=0.447; *F*(2,14)=5.64, *p*=0.016) being increased in magnitude for the least severe and most severely affected patients compared to those in the middle range of severity. However, this correlation zwas not deemed to be significant when a correction was applied for multiple statistical comparisons (Bon Ferroni corrected alpha of 0.0031).

## 3 Discussion of Experiment 1

The key findings of Experiment 1 are that PDs were unimpaired at switching between conflicting stimulus-saccade mappings in terms of the magnitude of the “switch cost” on response errors and saccade latencies following a change in rule mappings. However, PDs made increased uncorrected response errors overall in switching blocks and were also slower than controls at learning the first cue-saccade rule in the initial uniform rule block. These finding are interesting in the light of previous studies which have examined task switching in PD as well as earlier studies using the oculomotor rule switching task in other patient populations.

Cools et al. (2001a) attempted to disambiguate the nature of the Parkinsonian deficit in cognitive flexibility by dissociating the attentional set switching, rule switching and working memory components of classic cognitive switching tasks. This was achieved by explicitly cuing task rules (eliminating the working memory component) and presenting response imperative stimuli which could either be in conflict with both, or exclusively associated with one of the two possible task rules (isolating the response/attentional conflict component). It was concluded that the deficit in PD arose during rule switching only under conditions of conflict and could not be explained by other factors such as working memory load. However, in the rule switching task used here there is always rule conflict as cues can indicate a left or a right response dependent upon the rule. The test also places demands on working memory and attentional selection processes as the current rule has to be held in mind and distracting information (e.g. the alternate rule) needs to be ignored. In this sense the absence of a clear switching deficit in the PD group is surprising.

More recent work has extended Cools et al.’s original findings and has further clarified the conditions under which mild to moderate PDs show a deficit in task switching. Kehagia, Cools, Barker and Robbins (2009) and Pollux (2004) both used procedures which manipulated the demand to select between task relevant visual information on each trial as well as switching between stimulus-response mappings associated with the same visual cue. Deficient performance was only seen under conditions for which task irrelevant visual stimuli had to be ignored, whereas simple switching between two mutually incompatible stimulus-response mappings was found to be relatively normal in early stage medicated patients (i.e. a result compatible with the findings reported here). Kehagia concluded that dopaminergic striatal dysfunction (for which early stage PD seems a reasonable model) does not lead to a deficit in task rule switching per se, but does impair the ability to filter out task irrelevant perceptual input. The discrepancy with earlier findings may be due to the severity and heterogeneity of patients tested in previous studies with only more severely affected patients having a clear impairment in pure stimulus-response rule switching (Kehagia et al., 2009).

The performance of the mild medicated PDs described here constitutes a double dissociation in the oculomotor rule switching task when compared to impairments seen following inferior frontal cortical damage, in which initial rule learning was found to be intact, but performance in the rule switching blocks was markedly impaired (Hodgson et al., 2007). If it is assumed that PDs' suffer from a disruption to fronto-striatal circuitry, then these findings imply that damage to this network does not lead to identical sequelae regardless of the site of disruption, but instead can produce specific effects dependent upon whether cortical or sub-cortical components are affected. The performance of PDs also has interesting differences and similarities when compared to a patient with a well circumscribed supplementary eye field lesion who has been tested using the same task (Husain et al., 2003; Parton et al., 2007). Unlike PDs, this individual made a greatly increased number of corrected saccade errors on the trials immediately following rule changes, but showed slower learning of novel stimulus-saccade associations (Parton et al., 2007). It is possible that forming new arbitrary associations between stimuli and saccades is a key function of the cortico-striatal circuit linking the supplementary eye fields with the striatum, with damage to either component of the circuit in this case leading to impairments in this ability.

Another aspect of the current task which has been investigated in patients elsewhere is the slowing of responses directed back to locations at which negative feedbacks have been presented on the previous trial. This reward dependent negative priming effect (Tipper, 1985) is reduced in magnitude following orbitofrontal/ventro-medial prefrontal damage (Hodgson et al., 2002). Schizophrenic patients have also been shown to have an enhanced bias to respond *away* from negative feedbacks in the task relative to controls (Huddy et al., 2011). In this study the effect was found to be reduced in magnitude in the PD group (Fig. 4) and did not reach statistical significance, whilst still being strongly significant for control participants. However, the 2-way group by negative priming interaction did not reach statistical significance, suggesting that the magnitude of the effect was more variable rather than being consistently reduced across patients.

Issues of statistical power need to be considered in relation to the absence of a significant interaction effect between Group and the Negative Priming effect. A key issue is whether the effect is *consistently* inconsistent independent of sample size for the PD group. In order to examine this, a simple “resampling” approach was used in which a randomly selected sub-set of controls and patients were analysed to assess whether a similar pattern was observed with fewer participants (7 controls and 9 patients chosen via the random case selection function within SPSS v19). This analysis once again revealed a highly significant priming effect overall in the reduced data set (*F*(1,12)=10.68, *p*=0.007) and an insignificant interaction effect between Group and the negative priming effect (although with a *higher F*-value than that found in the main analysis: *F*(1,12)=2.66 relative to *F*(1,30)=0.78). As in the main analysis however, the negative priming effect was found to be highly significant for controls (*F*(1,7)=12.72, *p*=0.009) but not for patients (*F*(1,5)=1.37, *p*=0.295) when the two groups of the sub-set were analysed separately. This suggests that the absence of a significant interaction between participant group and the negative priming effect reflects genuine hetereogeneity within the PD group.

In previous work we have used the term “inhibition of return” to refer to the response negative priming effect observed in the rule switching task. This phrase was originally coined by Posner et al. to describe an increase in response time to targets appearing at recently stimulated locations (Posner, Rafal, Choate, & Vaughan, 1985). However, several authors have speculated that the effect may be important in contexts far removed from the classical spatial cueing procedure. For example, its utility in controlling eye movements during search (Gilchrist & Harvey, 2000) or as a “foraging facilitator” has been suggested (Klein & MacInnes, 1999). Consistent with this idea our own previous studies has shown that a reduced inhibition of return/negative priming effect in the rule switching task is associated with disorganized/inefficient visual search strategies following orbitofrontal cortex damage (Hodgson et al., 2002). It is also interesting to note that recent work has shown that PDs have disordered eye movement search patterns characterized by increased re-fixations of targets and distractors during visual search (Mannan, Hodgson, Husain, & Kennard, 2008).

In summary, the results on the one hand are consistent with previous findings which have shown that stimulus-response rule switching performance is relatively intact in early stage PDs, whilst at the same time suggesting an impairment in learning novel stimulus-response mappings and a possible deficiency in processing of negative feedbacks dependent upon dopaminergic state. But once an initial rule has been learned patients appear to be able to utilize relatively intact cognitive representations of task rules to maintain and switch between related SR mappings as indicated by the lack of a significant difference in acquiring the first instance of the reversed rule in switching blocks (Fig. 2). If this is the case then PDs might be expected to be maximally impaired at tasks which placed enhanced demands on “trial and error” type learning and rely less on explicit representations of task rules. Experiment 2 was therefore designed to investigate the idea that there might be two types of learning, one based on explicit knowledge of rules, the other based on associative learning processes, and that the second of these mechanisms may be the one which is primarily affected in the early stages of PD. We compared patients and controls on a modified version of the rule switching task in which feedback was given stochastically such that a “correct” response only resulted in a positive feedback with 80% probability. In all other respects the task was closely matched to that used in Experiment 1.

## 4 Experiment 2

### 4.1 Methods

#### 4.1.1 Participants

The control group comprised 8 neurologically normal participants. 2 were female and 6 were female. Ages ranged from 50 to 70 years, with a mean age of 57 years. Eight patients with mild to moderate PD were recruited from the movement disorders clinic at the Royal Devon and Exeter Hospital. Six were male and 2 were female. Their ages ranged from 63 to 74, with a mean age of 62 years. Three of the control subjects and 3 of the PD patients had previously completed the simple rule reversal task in an earlier testing session.

#### 4.1.2 Task and procedure

The task and procedure were identical to those used in Experiment 1 accept for the following differences. Feedback given by the computer was only reliable on 80% of the trials, such that adehence to the currently correct rule would not reliably produce a correct feedback and execution of the “incorrect” response could produce a positive feedback on 20% of the trials (Fig. 1b). Participants were instructed that the computer used a rule linking the colour of the central cue with saccades to the left or right, but that the computer would sometimes “tell lies” such that it would only give you accurate feedback around 80% of the time. As before they were also told that the rule the computer was using could change “several times” during the course of the test and their task was to “find as many happy faces as possible”.

The central cue was always either yellow or blue in Experiment 2. The start of each new rule within the switching blocks was deemed to be the first trial after a negative feedback had been received following a change in rule (i.e. due to the probabilistic nature of the task a false positive feedback could occur by chance following a change in probabilistic contingencies such that it would have been impossible for the participant to know that the rule had changed). Based upon this criterion, rule changes occurred in a uniformly random distribution every 10–16 trials.

Participants completed an initial block of 80 trials in which the optimal response rule stayed the same throughout (*Probabilistic Association task*). They then completed two blocks of 80 trials in which the rule could change (*Probabilistic Reversal task*) with an average of 6 rule changes occurring in each block.

We use the terms “Rule” and “Errors” in the methods, results and discussion of Experiment 2 for consistency in relation to Experiment 1, although the probabilistic nature of the task means that there is always uncertainty associated with optimal stimulus-response-outcome mappings and a number of sub-optimal rule based strategies can be applied in the task (see “Strategic effects on saccade direction” below). Based upon the definition of a correct response as conforming to a response selected on the basis of an optimal rule, the “trials to criterion” measure used to measure speed of rule learning in the simple associative blocks (see Experiment 1 methods) was determined using the same criterion of 8 consecutive correct trials as was used in Experiment 1.

### 4.2 Results

#### 4.2.1 Probabilistic association

Independent sample, 2-tailed *t*-tests were used to compare the main performance measures between PDs and healthy control participants in the probabilistic association block in which participants had to learn a single stochastically reinforced colour-saccade association. The results showed that PDs had significantly elevated primary saccade latencies (*t*=2.65, d.f.=14, *p*=0.019) and reduced amplitude saccades in the task (*t*=3.90, 14, *p*=0.003) in the task, but there was no significant difference in error rates (*t*=1.25, *p*=0.23) or trials to criterion (*t*=1.36, *p*=0.195) (Fig. 2).

#### 4.2.2 Probabilistic rule switching

##### 4.2.2.1 Saccadic amplitude

Primary saccadic amplitude (i.e. size of first saccade on each trial) was analysed using a 2-way ANOVA with subject group (control/patient) and trial after rule change (1–8) as factors. PDs saccades showed significant saccadic hypometria relative to control participants (main effect of Group *F*(1,14)=4.65, *p<*0.05), but there was no effect of Trial (*F*(7,98)=1.14, *p*=0.16) or interaction between Trial and Group (*F*(7,98)=0.89, *p*=0.48).

##### 4.2.2.2 Saccade latencies

The latency of the first saccade following cue onset was analysed using a 2-way Analysis of variance with Group (control/patient) and Trial after rule change (1–8) as factors. This revealed a significant effect of Group (*F*(1,14)=5.81, *p*=0.04), with latencies being increased in PDs relative to controls. There was a significant main effect of trial (*F*(7,98)=3.45, *p*=0.002) but no interaction effect between Trial and Group (*F*(7,98)=0.80, *p*=0.59). Response latency on the trial immediately preceding the feedback which signalled a rule change (labelled trial 0 in Fig. 3) were also analysed and found not to differ significantly between groups (independent samples *t*-test *t*=1.52, d.f.=14, *p*=0.15).

##### 4.2.2.3 Errors

In the case of the probabilistic reversal task, “errors” were defined as saccadic responses which were directed towards the location specified by the sub-optimal rule. As in Experiment 1, a distinction was made between uncorrected and corrected errors for which an initial saccade in the “incorrect” direction was rapidly followed by a secondary saccade towards the alternate location.

A 2-way Analyses of variance (ANOVAs) were carried out on *total errors* (corrected and uncorrected) with Group (control/patient) and Trial after rule change (1–8) as factors. This revealed a significant effect of Trial (*F*(7,98)=2.78, *p*=0.011) across all participants and a significant effect of Group (*F*(1,12)=2.76, *p*=0.048) but no interaction between Trial and Group (*F*(7,98)=1.18). Total errors (based on the updated rule) on the trial for which participants received the feedback that indicated a rule change (i.e. trial 0) found to be significant *reduced* in the PD group (*t*=2.29, d.f.=14, *p*=0.038).

A similar analysis for *uncorrected errors* revealed a significant effect of Group (*F*(1,14)=7.17, *p*=0.018) as well as Trial (*F*(7,98)=3.57, *p*=0.002). The interaction between Trial and Group was also significant when the first trial following a rule change was compared to subsequent trials (*F*(2,13)=5.34, *p*=0.02). Means comparisons revealed that the difference in error rates between groups was only significant for responses more than 3 trials following a rule change (independent sample *t*-test *t*=2.73, d.f.=14, *p<*0.02), with no significant difference apparent when the analysis was confined to responses on the 1st to 3rd trial after the rule change (*t*=0.137, *p*=0.893) (Fig. 3b). As with total errors, analysis of uncorrected errors on Trial 0 showed a trend towards reduced errors in the PD group, but no significant difference between groups (*t*=1.53, d.f.=14, *p*=0.05).

Analysis of *corrected errors* (expressed as a percentage of all trials) showed no significant difference between Group (*F*(1,14)=0.568, *p*=0.46), Trial after rule change (*F*(7,98)=1.27, *p*=0.274) or interaction between Group and Trial (*F*(7,98)=0.416, *p*=0.89), indicating that PDs were no more or less likely than controls to make corrected response errors. There was no significant difference in corrected errors for Trial 0 between the two groups (*t*=1.25, d.f.=14, *p<*0.23).

##### 4.2.2.4 Negative primiing effect

A 3-way ANOVA with Type of feedback on previous trial (positive/negative), Direction of response (Same/Different to last trial) and Group (PD/Control) was used to assess whether a feedback modulated negative priming effect was present with probabilistic feedback. Although latencies were found to be significantly slower for trials immediately following a negative feedback (*F*(1,14)=11.62, *p<*0.005), this effect did not interact with the direction of response (*F*(1,14)=1.89, *p*=0.191) and there were no higher order interaction effects involving Group.

##### 4.2.2.5 Correlations with disease severity

We examined whether performance measures were correlated with disease severity using linear and quadratic fit functions. Using a standard alpha value of 0.05 to assess significance there was a significant positive linear relationship between disease severity and the number of corrective saccade errors (*F*(1,6)=9.48, *p*=0.022) and a significant linear correlation between mean response latency overall, with response latency *decreasing* with UPDRS/Webster score (*F*(1,6)=7.01, *p*=0.038). However, none of the correlations were judged to be significant based upon a Bon Ferroni corrected significance threshold of *p*=0.0031.

##### 4.2.2.6 Strategic effects on saccade direction

Two possible explanations for the increased error rates in patients relative to controls would be that they were either updating rules too frequently (e.g. following each negative feedback received) or not frequently enough (e.g. using the same response rule throughout a block). In order to test this we examined whether subjects used the same or a different rule to determine the direction of their saccadic response on the current relative to the last trial, dependent upon whether the last feedback was either positive or negative. This analysis showed that, based upon the direction of the primary saccade executed by participants on each trial, rules were updated more frequently following a negative relative to a positive feedback (15% following +ve relative to 46% following –ve feedback overall: *F*(1,14)=45.10, *p<*0.001), but the rate of spontaneous rule updating between the two groups did not differ (Con: 27% versus PDs: 33%; *F*(1,14)=0.861, *p*=0.37), neither was there interaction between group and feedback type on the degree to which the rule was updated (10% of trials in Controls versus 20% in PDs following +ve feedback; 45% in Controls and 47% in PDs following −ve feedback; *F*(1,14)=0.671, *p*=0.43).

Participants also showed evidence for use of response alternation strategy, such that they were more likely to make saccades opposite to the location they had selected on the preceding trial (*F*(1,12)=13.87, *p*=0.002; Same direction: 40.8%; Different: 59.8%). Unlike Experiment 1 however, this bias was not dependent upon the type of feedback received on the last trial (positive versus negative) (57% versus 61% different direction responses on positive versus negative feedback trials respectively; *F*(1,14)=1.07, *p*=0.32), neither was there a difference in the use of this sub-optimal strategy across the two participant groups (main effect of group: *F*(1,14)=2.11, *p*=0.17; interaction between group and feedback type: *F*(1,14)=1.065, *p*=0.32).

## 5 General discussion

The key finding of Experiment 2 was that PDs made increased errors and longer response latencies relative to controls when switching between stimulus-saccade associations which were subject to stochastic reinforcement. Several possible accounts will be considered which might explain the findings of the two experiments together.

The first possibility is that PDs' deficit in learning and maintaining stimulus-response mappings is a side effect of dopaminergic medication. Recent computational simulations have suggested that dopaminergic medication could affect associative learning processes (Chakravarthy et al., 2010; Frank, 2005; Graybiel, 1997; Schultz, Dayan and Montague, 1997) and other work has also suggested that mild medicated but not un-medicated patients show deficits in monitoring probabilistic contingenices (Swainson et al., 2000). Such a medication effect would be of interest both in terms of its implications for people with Parkinsons, as well as for our understanding of the function of dopamine within cortico-striatal circuitry. Ethical constraints precluded us pursuing a direct comparison of patients on and off medication, but even if we had been able to do so the relationship between dopaminergic state and performance is such that a simple On/Off medication comparison would not provide a conclusive test of a medication based explanation (Cools et al., 2001c; Rowe et al., 2008).

An alternative view of the results is that they reflect a disease related impairment in stimulus-induced distractibility across all PD patients under conditions where one stimulus has to be attended to whilst another has to be ignored (Kehagia et al., 2009; Pollux, 2004). This is the case in the current tasks where participants need to ignore distracting negative feedbacks and act on the basis of the rule they know to be correct. However, analysis of the pattern of errors in both Experiment 1 and 2 showed that patients were no more likely than controls to make a response error away from the location of a negative feedback on the previous trial, suggesting that they were not overly distracted by negative feedbacks. Furthermore, analysis of strategic factors affecting saccade direction in Experiment 2 showed that both groups made use of sub-optimal response and rule alternation strategies, whereby they spontaneously changed response rules or selected the alternate response direction relative to the preceding trial. However, the rate of spontaneous rule updating and response alternation following negative feedbacks did not differ between the two groups as might be expected if PDs were more distracted relative to controls.

Another possibility is that the increase in errors observed in PDs in both experiments is due to anticipatory updating of rules. The increase in uncorrected errors for PD patients was largely confined to later trials following a rule change in Experiment 1. In the case of the probabilistic task this would predict increased errors in PDs following the occurrence of “false” negative feedbacks. However, this was not found top be the case (see above and *Experiment 2*, *Results: Strategic effects on saccade direction*). At the same time, the significant increase in latencies for responses on trials occurring more than 6 trials after a rule change in Experiment 2 (Fig. 2b) might be seen as consistent with participants generating an expectation or anticipating an upcoming rule change. For both experiments the significant increase in errors for PD patients was due primarily to the occurrence of errors on later trials (rather than those immediately following a rule change). This pattern would be consistent with increased anticipatory updating of rules in the PD group or failures to maintain rules in working memory (i.e. loss of set).

As fewer participants were used in Experiment 2 relative to Experiment 1 it is possible that differences in statistical power might explain apparent dissociations in performance between the two versions of the task. In particular, the absence of a significant difference in performance between patients and controls in the Probabilistic Association rule learning block for Experiment 2 (Fig. 2) should be interpreted with caution. Taking a similar resampling approach to that outlined in the discussion of power effects for Experiment 1 (see above), analysis of a randomly selected sub-sample of participants from Experiment 1 (8 patients and 8 control participants) revealed insignificant differences in errors (*t*=1.83, *p*=0.089) and trials to criterion (*t*=1.52, *p*=0.15) for the Simple Association task block. A selective impairment in PD for learning single rules under conditions of reliable relative to probabilistic feedback therefore seems unlikely as well as being theoretically implausible (as both tasks would engage similar mechanisms responsible for trial and error learning). However, the issue of reduced participants numbers in Experiment 2 cannot easily explain the key finding in relation to switching blocks. Significant interaction effects between Trial and Group were apparent in switching blocks with *fewer* patients in Experiment 2 (8 patients), but were absent with the larger sample size available in Experiment 1 (16 patients).

Another explanation for the lack of a significant difference between patient and controls in the probabilistic association block (Experiment 2) is the increased demands placed on control subjects, rather than relatively enhanced performance in patients. Although well matched with the non-probablistic rule switching procedure in terms of visuo-motor characteristics, the probabilistic rule switching task (Experiment 2) has some important differences which are worthy of discussion. Learning rules with stochastic feedback implicitly demands integration of cue–response–outcome relationships over the course of several trials. It is not possible for rule changes to be reliably detected based upon evidence accumulated in a single trial, such that a clearly defined single trial “switch cost” would not be expected in the probabilistic version of the task. A cost is observed on response errors for control participants (but not PDs) following rule changes in Experiment 2 reflecting increased certainty regarding the correct rule as the number of trials after a rule switch increases. However, as any given negative feedback has a 20% chance of being a “false” error in the task, even a perfect observer needs to integrate information over at least 2 consecutive trials before establishing with 95% certainty the “correct” rule. The lack of a significant increase in response latencies immediately following a rule change also suggests that the cognitive demands of the task may be more continuous in probabilistic rule switching relative to the standard version of the task.

A consistent finding between Experiment 1 and 2 was that patients' saccadic eye movements were hypometric relative to controls. Previously, Parkinsonian hypometria has only been found consistently under “open loop” or memory-guided conditions where peripheral target stimuli are not present. Responding in stimulus driven conditions is typically found to be normal or even show evidence for speeding of response latencies under certain conditions in PD (Chambers & Prescott, 2010). The present experiments suggest that hypometria may occur under other conditions where saccades have to be made partially on the basis of internal cognitive representations (in this case learned rules or stimulus response mappings) even when visual markers for responding are provided in the display. Hypometria in saccadic movements when they have to be executed based upon cognitive representations is also consistent with the theory that the dorsal striatum is involved in the release of goal directed acts, rather than habitual or stimulus elicited behaviour (Grahn et al., 2008).

Interestingly, Parkinsonian like hypometria in memory-guided saccades has also been reported following supplementary motor area damage, alongside impaired learning of novel stimulus-saccade associations (Parton et al., 2007). One possibility is that rather than reflecting abnormal function of a putative frontal eye field-caudate nucleus circuit or a medication induced dysfunction within striatal associative learning systems, both hypometria and rule learning impairments in PD instead represent disease related dysfunction to a dorso-medial frontal-putamen circuit (Alexander & Crutcher, 1990; Grahn et al., 2008). Interestingly, very recent combined fMRI and DTI imaging studies have questioned the existence of a dedicated oculomotor loop via the caudate nucleus and have instead highlighted an oculomotor role for the putamen in the control of volitional saccades (Neggers et al., 2012).

Another aspect of oculomotor function which has often been claimed to be impaired in PD is inhibitory suppression of movements elicited by a peripheral stimulus onset (Briand et al., 1999; Chan, Armstrong, Pari, Riopelle, & Munoz, 2005; Fukushima et al., 1994; van Koningsbruggen, Pender, Machado, & Rafal,, 2009; Lueck et al., 1990). Significantly increased corrected saccades in the rule switching task following a change in stimulus-response mappings have also been interpreted as representing failures to inhibit saccades in response to a centrally presented cue based upon recently active rules/associations (Hodgson et al., 2004, 2007; Husain et al., 2003). Based upon this measure of inhibitory control however, the present study found no evidence for an impairment in this type of inhibitory control in mild medicated PDs.

## 6 Summary and conclusions

The results show that mild to moderately affected people with Parkinsons disease (PDs) are impaired in tasks which require learning of novel stimulus-saccade mappings or switching between stochastically reinforced mappings. When the stimulus-saccade rules underlying a task are more transparent, PDs show normal response times and error rates. These findings are interpreted as evidence for an impairment in associative learning processes in PDs, accompanied by relatively intact cognitive representations of task rules.

Further research could investigate how these findings might relate to performance of everyday tasks which require coordination of arbitrary mappings between perceptual cues and saccadic responses (Land & Furneax, 1997; Poliakoff & Smith Spark, 2008; Tatler, Hayhoe, Land, & Ballard, 2011). One prediction is that mild medicated PDs should have more difficulty learning new visuo-motor tasks and skills (e.g. learning to cook a new recipe or operate a new computer programme) than they would in performing and switching between routine tasks that have already been learned.

## Figures and Tables

**Table 1:** Patient details. Exp1=Simple Associative Task and Rule Switching Tasks completed. Exp1a=Simple Associative Task only completed. CX=Charing Cross Hospital recruitment; EX=Royal Devon and Exeter Hospital. H&Y=Hoen and Yahr sore; MMSE=Mini mental state assessment; NART=National Adult Reading Test errors;UPDRS=Unified Parkinsons Disease Rating Scale.

**Patient**	**Tasks completed**	**Recruitment site**	**Age**	**Sex**	**Medication**	**Disease duration**	**Webster (UPDRS III)**	**H&Y**	**MMSE**	**NART**	**Digit Span (total correct)**
01	Exp1	CX	72	F	Sinemet CR; Pramipexole	6 years	13 (19)	1.5	30	9	15
02	Exp1	CX	66	M	Sinemet CR	7 years	23 (33)	3	29	19	14
03	Exp1a	CX	56	M	Sinemet; Sinemet CR; Cabergoline	9 years	24 (34)	3	23	5	13
04	Exp1	CX	69	M	Benhexol; Selegilin;	10 years	5 (7)	1	30	8	15
05	Exp1	CX	54	F	Sinamet CR; Sinemet plus; pergoline	5 years	16 (23)	2.5	30	3	15
06	Exp1a	CX	74	M	Sinemet; Sinemet CR	13 years	19 (27)	4	26	15	14
07	Exp1	CX	63	F	Sinemet; Pergolide	8 years	14 (20)	2.5	25	12	17
08	Exp1	CX	54	F	Madopar; Selegiline; Propanolol	6 years	14 (20)	2	28	21	12
09	Exp1	CX	57	M	Sinemet	6 years	15 (21)	2.5	29	7	18
10	Exp1	CX	45	M	Benhexol; Cabergoline	5 years	11 (16)	2	27	11	18
11	Exp1	CX	61	F	Sinemet; Benhexol	1 years	2 (3)	1	30	8	22
12	Exp1	CX	56	F	Pramipexole	6 years	13 (19)	1.5	30	20	18
13	Exp1	CX	48	M	Sinemet CR; Pergolide	4 years	26 (37)	3	27	24	16
14	Exp1a; Exp2	EX	74	F	Madopar; Pramipexole	7 years	5 (7)	1	30	14	15
15	Exp1; Exp2	EX	71	M	Madopar	18 months	8 (11)	2	29	13	15
16	Exp1	EX	74	M	Selegiline; Pramipexole	4 Years	12.5 (18)	2	29	9	15
17	Exp1; Exp2	EX	72	M	Pramiprexole; Sinemet	6 years	11 (16)	2	27	5	12
18	Exp1	EX	70	F	Sinemet CR; Madopar	16 years	15 (21)	2	30	5	13
19	Exp2	EX	74	M	Pramipexo	1 year	9 (13)	2	27	5	15
20	Exp2	EX	66	M	Sinemet	6 years	11 (16)	2	30	6	13
21	Exp1a	EX	79	F	Sinemet; selegiline	7 years	11 (16)	2.5	28	12	16
22	Exp2	EX	65	M	Pramipexole	4 years	15 (21)	2.5	29	6	22
23	Exp1	EX	71	M	Madopar	18 months	11 (16)	2.5	28	10	13
24	Exp1; Exp2	EX	72	F	Sinemet CR; Selegiline; Amantadine	7–8 years	11 (16)	2	27	5	15
25	Exp 2	EX	63	M	Sinemet CR; pramipexole	13 years	11 (16)	2	26	10	11

## References

[bib1] Alberts J.L., Saling M., Adler C.H., Stelmach G.E. (2000). Disruptions in the reach-to-grasp actions of Parkinson's patients. Experimental Brain Research.

[bib2] Alexander G.E., Crutcher M.D. (1990). Functional architecture of basal ganglia circuits: Neural substrates of parallel processing. Trends in Neurosciences.

[bib3] Bowen F.P., Kameinny R.S., Burns M.M., Yahr M. (1975). Parkinsonism: Effects of levodopa treatment on concept formation. Neurology.

[bib4] Briand K.A., Strallow D., Hening W., Poizner H., Sereno A.B. (1999). Control of voluntary and reflexive saccades in Parkinson's disease. Experimental Brain Research.

[bib5] Cameron I.G.M., Pari G., Alahyane N., Brien D.C., Coe B.C., Stroman P.W., Munoz D.P. (2012). Impaired executive function signals in motor brain regions in Parkinson's disease. Neuroimage.

[bib6] Chakravarthy V.S., Joseph D., Bapi R.S. (2010). What do the basal ganglia do? A modeling perspective. Biological Cybernetics.

[bib7] Chambers J.M., Prescott T.J. (2010). Response times for visually guided saccades in persons with Parkinson's disease: A meta-analytic review. Neuropsychologia.

[bib8] Chan F., Armstrong I.T., Pari G., Riopelle R.J., Munoz D.P. (2005). Deficits in saccadic eye-movement control in Parkinson's disease. Neuropsychologia.

[bib9] Cools A.R., Van den Bercken J.H.L., Horstink M.W.I., van Spaendonck K.P.M., Berger H.J.C. (1984). Cognitive and motor shifting aptitude disorder in Parkinson's disease. Journal of Neurology, Neurosurgery, and Psychiatry.

[bib10] Cools R., Barker R.A., Sahakian B.J., Robbins T.W. (2001). Mechanims of cognitive set flexibility in Parkinson's disease. Brain.

[bib11] Cools R., Barker R., Sahakian B.J., Robbins T.W. (2001). Enhanced or impaired cognitive function in Parkinson's disease as a function of dopaminergic medication and task demands. Cerebral Cortex.

[bib12] Cools R., Barker R.A., Sahakian B.J., Robbins T.W. (2001). Enhanced or impaired cognitive function in Parkinson's disease as a function of dopaminergic medication and task demands. Cerebral Cortex.

[bib14] Crawford T.J., Henderson L., Kennard C. (1989). Abnormalities of non-visual guided eye movements in Parkinson's disease. Brain.

[bib15] Damier P., Hirsch E.C., Agid Y., Graybiel A.M. (1999). The substantia nigra of the human brain II. Patterns of loss of dopamine-containing neurons in Parkinson's disease. Brain.

[bib16] Downes J.J., Roberts A.C., Sahakian B.J., Evenden J.L., Morris R.G., Robbins T.W. (1989). Impaired extra-dimensional shift perform-ance in medicated and unmedicated Parkinson's disease: Evidence for a specific attentional dysfunction. Neuropsychologia.

[bib17] Fahn S., Elton R.L., Fahn S., Marsden C.D., Goldstein M., Calne D.B. (1987). UPDRS program members. Unified Parkinsons Disease Rating Scale. Recent developments in Parkinsons disease (Vol. 2).

[bib18] Flowers K.A., Robertson C. (1985). The effect of Parkinson's disease on the ability to maintain a mental set. Journal of Neurology, Neurosurgery and Psychiatry.

[bib19] Frank M.J. (2005). Dynamic dopamine modulation in the basal ganglia: A neurocomputational account of cognitive deficits in medicated and nonmedicated Parkinsonism. Journal of Cognitive Neuroscience.

[bib20] Fukushima J., Fukushima K., Miyasaka K., Yamashita I. (1994). Voluntary control of saccadic eye movement in patients with frontal cortical lesions and Parkinsonian patients in comparison with that in schiophrenics. Biological Psychiatry.

[bib21] Gilchrist I.D., Harvey M. (2000). Refixation frequency and memory mechanisms in visual search. Current Biology.

[bib22] Golding C.V.P., Danchaivijitr C., Hodgson T.L., Tabrizi S.J., Kennard C. (2006). Identification of an oculomotor biomarker of preclinical Huntington disease. Neurology.

[bib23] Grahn J.A., Parkinson J.A., Owen A.M. (2008). The cognitive functions of the caudate nucleus. Progress in Neurobiology.

[bib24] Grant D.A., Berg E.A. (1948). A behavioral analysis of degree of reinforcement and ease of shifting to new responses in a weigl-type card-sorting problems. Journal of Experimental Psychology.

[bib25] Graybiel A.M. (1997). The basal ganglia and cognitive pattern generators. Schizophrenia Bulletin.

[bib26] Hodgson T., Chamberlain M., Parris B., James M., Gutowski N., Husain M, Kennard C. (2007). The role of the ventrolateral frontal cortex in inhibitory oculomotor control. Brain.

[bib27] Hodgson T.L., Golding C., Hyona J., Radach R., Deubel H. (2003). Executive contributions to eye movement control. The minds eye: Cognitive and applied aspects of eye movement research.

[bib28] Hodgson T.L., Mort D.J., Chamberlain M.M., O'Neill K., Kennard C. (2002). Orbitofrontal cortex mediates inhibition of return. Neuropsychologia.

[bib29] Hodgson T.L., Dittrich W., Henderson L., Kennard C. (1999). Eye movements and spatial working memory in Parkinson's disease. Neuropsychologia.

[bib31] Hodgson T.L., Golding C., Molyva D., Rosenthal C., Kennard C. (2004). Eye movements during task switching: Reflexive, symbolic, and affective contributions to response selection. Journal of Cognitive Neuroscience.

[bib30] Hoehn M.M., Yahr M.D. (1967). Parkinsonism: Onset, progression and mortality.

[bib32] Huddy V.C., Hodgson T.L., Ron M.A., Barnes T.R.E., Joyce E.M. (2011). Abnormal negative feedback processing in first episode schizophrenia: Evidence from an oculomotor rule switching task. Psychological Medicine.

[bib33] Husain M., Parton A., Hodgson T., Mort D., Rees G. (2003). Self-control during response conflict by human supplementary eye field. Nature Neuroscience.

[bib34] Jackson S.R., Jackson G.M., Harrison J., Henderson L., Kennard C. (1995). The internal control of action and Parkinsons-disease—a kinematic analysis of visually-guided and memory-guided prehension movements. Experimental Brain Research.

[bib35] Kehagia A.A., Cools R., Barker R.A., Robbins T.W. (2009). Switching between abstract rules reflects disease severity but not dopaminergic status in Parkinson's disease. Neuropsychologia.

[bib36] Ketcham C.J., Hodgson T.L., Kennard C., Stelmach G.E. (2003). Memory-motor transformations are impaired in Parkinson's disease. Experimental Brain Research.

[bib37] Kish S.J., Shannak K., Hornykiewicz O. (1988). Uneven patterns of dopamine loss in the striatum of patients with idiopathic Parkinson's disease. Pathophysiologic and clinical implications. New England Journal of Medicine.

[bib38] Klein R.M., MacInnes W.J. (1999). Inhibition of return is a foraging facilitator in visual search. Psychological Science.

[bib40] Land M.F., Furneax S. (1997). The knowledge base of the oculomotor system. Philosophical transactions of the Royal Society of London B.

[bib41] Lehericy S, Ducros M, Van de Moortele P.F., Francois C., Thivard L., Poupon C. (2004). Diffusion tensor fiber tracking shows distinct corticostriatal circuits in humans. Annals of Neurology.

[bib42] Levy R., Friedman H.R., Davachi L., Goldman-Rakic P.S. (1997). Differential activation of the caudate nucleus in primates performing spatial and nonspatial working memory tasks. Journal of Neuroscience.

[bib43] Lueck C.J., Tanyeri S., Crawford T.J., Henderson L., Kennard C. (1990). Antisaccades and remembered saccades in Parkinson’s disease. Journal of Neurology, Neurosurgery and Psychiatry.

[bib39] van Koningsbruggen M.G., Pender T., Machado L., Rafal R.D. (2009). Impaired control of the oculomotor reflexes in Parkinson's disease. Neuropsychologia.

[bib44] Mannan S.K., Hodgson T.L., Husain M., Kennard C. (2008). Eye movements in visual search indicate impaired saliency processing in Parkinson’s disease. Progress Brain Research.

[bib45] Middleton F.A., Strick P.L. (2000). Basal ganglia and cerebellar loops: Motor and cognitive circuits. Brain Research Brain Research Review.

[bib46] Mosimann U.P., Muri R.M., Burn D.J., Felblinger J., O’Brien J.T., McKeith I.G. (2005). Saccadic eye movement changes in Parkinson’s dementia and dementia with Lewy bodies. Brain.

[bib847] Neggers, S. F. W., Van Diepen, R. M., Zandbelt, B. B., Vink, M., Mandl, R. C. W., & Gulteling, T. P. (2012). A functional and structural investigation of the human fronto-basal volintional saccade network. *PloS One*, 7(1), e29517, http://dx.doi.org/10.1371/journal.pone.0029517. 10.1371/journal.pone.0029517 PMC3250458 22235303

[bib47] Owen A.M., James M., Leigh P.N., Summers B.A., Marsden C.D., Quinn N.P., Lange K.W., Robbins T.W. (1992). Fronto-striatal cognitive deficits at different stages of Parkinson’s disease. Brain.

[bib48] Owen A.M., Roberts A.C., Hodges J.R., Summers B.A., Polkey C.E., Robbins T.W. (1993). Contrasting mechanisms of impaired attentional set-shifting in patients with frontal lobe damage or Parkinson’s disease. Brain.

[bib49] Parton A., Nachev P., Hodgson T.L., Mort D., Thomas D., Ordidge R., Morgan P.S., Jackson S., Rees G., Husain M. (2007). Role of the human supplementary eye field in the control of saccadic eye movements. Neuropsychologia.

[bib50] Poliakoff E, Smith Spark JH (2008). Everyday cognitive failures and memory problems in Parkinson’s patients without dementia. Brain and Cognition.

[bib51] Pollux P.M.J. (2004). Advance preparation of set-switches in Parkinson’s disease. Neuropsychologia.

[bib52] Posner M.I., Rafal R.D., Choate L.S., Vaughan J. (1985). Inhibition of return: Neural basis and function. Cognitive Neuropsychology.

[bib53] Postle B.R., D’Esposito M. (1999). Dissociation of human caudate nucleus activity in spatial and nonspatial working memory: An event-related fMRI study. Cognitive Brain Research.

[bib54] Rand M.K., Stelmach G.E. (2000). Movement accuracy constraints in Parkinson’s disease patients. Experimental Brain Research.

[bib55] Robert M.P.A., Nachev P.C., Hicks S.L., Golding C.V.P., Tabrizi S.J., Kennard C., Strupp M., Buttner U., Cohen B. (2009). Saccadometry of conditional rules in presymptomatic Huntington’s disease.

[bib56] Rogers R.D., Tipper S.P., Sahakian B.J., Hodges J.R., Polkey C.E., Kennard C., Robbins T.W. (1998). Dissociating executive mechanisms of task control following frontal lobe damage and Parkinsons disease. Brain.

[bib57] Rowe J.B., Hughes L., Ghosh B.C.P., Eckstein D., Williams-Gray C.H., Fallon S, Barker R.A., Owen A.M. (2008). Parkinson’s disease and dopaminergic therapy-differential effects on movement, reward and cognition. Brain.

[bib58] Schultz W., Dayan P., Montague P.R. (1997). A neural substrate of prediction and reward. Science.

[bib59] Swainson R., Rogers R.D., Sahakian B., Summers B., Polkey C., Robbins T.W. (2000). Probabilistic learningand reversal deficits in patients with Parkinson’s disease or frontal or temporal lobe lesions: Possible adverse effects of dopaminergic medication. Neuropsychologia.

[bib60] Swinnen S.P., Steyvers M., VanDenBergh L., Stelmach G.E. (2000). Motor learning and Parkinson’s disease: Refinement of within-limb and between-limb coordination as a result of practice. Behavioral Brain Research.

[bib61] Tatler B.W., Hayhoe M.M., Land M.F., Ballard D.A. (2011). Eye guidance in natural vision: Reinterpreting salience. Journal of Vision.

[bib62] Tipper S.P. (1985). The negative priming effect—Inhibitory priming by ignored objects. Quarterly Journal of Experimental Psychology Section A—Human Experimental Psychology.

[bib63] Webster D.D. (1968). Critical analysis of the disability in Parkinson’s disease. Modern Treatment.

